# Preference-Matched Multitask Assignment for Group Socialization under Mobile Crowdsensing

**DOI:** 10.3390/s23042275

**Published:** 2023-02-17

**Authors:** Mingyuan Zhang, Shiyong Chen, Zihao Wei, Yucheng Wu

**Affiliations:** School of Microelectronics and Communication Engineering, Chongqing University, Chongqing 400044, China

**Keywords:** mobile crowdsensing, heterogeneous multitasking, group-oriented, preference matching

## Abstract

Mobile crowdsensing (MCS) has been an emerging sensing paradigm in recent years, which uses a sensing platform for real-time processing to support various services for the Internet of Things (IoT) and promote the development of IoT. As an important component of MCS, how to design task assignment algorithms to cope with the coexistence of multiple concurrent heterogeneous tasks in group-oriented social relationships while satisfying the impact of users’ preferences on heterogeneous multitask assignment and solving the preference matching problem under heterogeneous tasks, is one of the most pressing issues. In this paper, a new algorithm, group-oriented adjustable bidding task assignment (GO-ABTA), is considered to solve the group-oriented bilateral preference-matching problem. First, the initial leaders and their collaborative groups in the social network are selected by group-oriented collaboration, and then the preference assignment of task requesters and users is modeled as a stable preference-matching problem. Then, a tunable bidding task assignment process is completed based on preference matching under budget constraints. Finally, the individual reasonableness, stability, and convergence of the proposed algorithm are demonstrated. The effectiveness of the proposed algorithm and its superiority to other algorithms are verified by simulation results.

## 1. Introduction

In recent years, the popularity and rapid development of smart devices (cell phones, tablets, sports bracelets, etc.) and the breakthroughs achieved by smart hardware have led to the integration of numerous powerful perceptrons in end devices, such as AI chips, cameras, microphones, etc. [1]. The Internet of Things (IoT) connects devices and equipment through sensing technology to achieve a leap forward in interconnecting things and objects [2], driving the development of mobile crowdsensing (MCS) under a large-scale sensing network [3]. As the development of MCS advances, researchers have come to acknowledge that it can significantly enhance the current approach of processes such as environmental monitoring and sensing [4,5]. Thus, investigating MCS technology is imperative. Essentially, MCS is a new mobile computing paradigm that combines IoT and perception technologies: from the perception point of view, the mobile crowdsensing network divides the perception task into several subtasks, which are then assigned to a large number of mobile users in the perception area for perception completion [6]. These mobile entities can be human beings utilizing intelligent devices, as demonstrated in this paper, or drones, or a coordination between human beings and drones for perception purposes [7].

The framework of a mobile group intelligence sensing system mainly contains three parts: task requester, user and sensing platform. The platform or allocator assigns the sensing tasks to the participating users for completion, and the data collection is usually uploaded by the users back to the platform and the allocator, who does the integration analysis and statistical processing after collecting the data [8,9,10,11]. Despite the numerous benefits of MCS, the following must be considered to build a sustainable mobile group intelligence sensing system: task assignment, incentive mechanisms, and user privacy and security [12,13]. Current research mainly focuses on either a single objective or the integration of multiple objectives, such as combining incentive mechanisms with privacy protection [14]. This paper particularly concentrates on task allocation.

In mobile group intelligence perception, the conflicting goals of task requesters to maximize the quality of task completion within budget constraints and participants to receive as many rewards as possible lead to a mismatch between task requesters and users and affect their motivation. Most existing studies only consider optimizing system-level goals, ignoring the needs and preferences of individual users. However, in a real mobile crowdsensing environment, maximizing only the perceived quality [15], social welfare [16], minimizing task cost [17] etc., users are not willing to give up their preferences to maximize the system utility. Therefore, task assignment without considering user preferences can lead to user dissatisfaction and affect future user engagement [18]. In addition, most existing studies only focus on recruiting a sufficient number of independent individual users to complete the task or simply clustering independent users based on task preferences [19], without taking into account the relationships among users during the recruitment process, which can impose high communication costs on the task [20]. However, in real-world mobile crowdsensing environments, users are not independent individuals but groups connected through social relationships, and users are more willing to cooperate with social partners, especially in tasks that require privacy sharing. In addition, closer social ties will accomplish collaborative tasks more effectively without incurring significant communication costs [21,22,23,24,25].

To solve the task assignment problem in group social scenarios where users have preferences, a heterogeneous task assignment mechanism under group grouping is described based on stable matching, and user recruitment is accomplished for the MCS system using many-to-many bilateral matching. The main contributions of this paper are summarized as follows.

- The concept of influence increase is introduced to describe the selection of grouping in the group social relations of the MCS system. When the grouping of users is completed, the influence increase will result in a more even distribution of affected users, thus increasing the diversity of users involved in the task.
- In order to reduce the cost of user selection, a collaborative group selection algorithm based on leader initialization is proposed to make the cost under user grouping significantly reduced based on group social user relationships.
- The task assignment problem in MCS systems is described as a bilateral matching problem with social relationships among users. To solve the problem, we propose a group-oriented adjustable bidding task assignment algorithm (GO-ABTA algorithm), which can transform the problem of preferences possessed by users into a distributed many-to-many resource trading problem. In addition, the algorithm ensures the individual rationality of both task and user and achieves the maximum utility of the system. The stability and convergence of the proposed algorithm, as well as its higher effectiveness compared to existing algorithms, are demonstrated through simulation experiments with real datasets, which improve the quality of task services.

The structure and sections of this paper are organized as follows. Section 2 describes the related work, Section 3 presents the system model and problem description, and Section 4 and Section 5 explain the proposed algorithm, respectively. Section 6 analyzes the experimental tests and results, and finally, a conclusion is presented in Section 7.

## 2. Related Works

The success of task assignment requires the involvement of a large number of users and aims at achieving a good trade-off between task quality and task cost. Inspired by the success of social networks (e.g., Facebook, WeChat, Twitter, etc.) in recent years, several studies [26,27,28,29,30] have proposed task assignment mechanisms that recruit users with the help of social networks. A novel task allocation algorithm is proposed in [26] that distributes sensory tasks fairly to users while using the Social Internet of Things (SIoT) to assess the reputation level of each member of the network. A dynamic task allocation algorithm for social recruiters was proposed in [27] to encourage users on the MCS platform to spread tasks through social networks and use social networks to recruit employees to complete tasks when the number of users is insufficient, while expanding the workforce. Location-based social networks (LBSNs) to obtain crowdfunding data are used in [28] to study the task allocation problem under influence maximization in LBSNs. A social network-based task allocation algorithm for mobile crowdfunding is considered in [29], rewarding users for providing perceptual data and their invitational behavior in mobile user social networks to select users with high perceptual power. Influence propagation on social networks to aid MCS user recruitment is used in [30], where task allocation is targeted to maximize coverage. A subset of users is first selected as the initial task participants, and then users who are influential and able to complete the task are recruited as staff. Most of the above research has focused on the assumption that at least one full candidate group provides enough candidate users to choose from (inconsistent with the real world), without considering the problem of collaborative multigroup task cooperation under the construction of compatible user groups with a large number of reachable users in social networks.

Recently, several works [31,32,33,34] used preference matching to solve the matching problem between task requesters and users, and stable matching theory was used in [31] to obtain satisfactory matches between users and task requesters, with two different stability conditions for user happiness defined. Finally, three efficient and stable task assignment algorithms are proposed and their stability under four different MCS scenarios is discussed. A distributed many-to-many matching model under task budget constraints was described in [32] by being constructed to describe the interaction between a perceptual task and a smartphone user. Then, a stable matching algorithm is designed to assign tasks to users and determine their rewards. Finally, the proposed algorithm is shown to have several desirable properties, including individual plausibility, stability and convergence. The impact of user preferences and the space-time characteristics of the task on the long-term utility of the employee is considered in [33] and a dynamic planning-based task allocation algorithm is proposed to ensure user satisfaction with the task. A task allocation algorithm based on stable matching under budget constraints is presented in [34], and rigorous theoretical analysis and simulation validation are given. Most of the above works focus on the assignment of homogeneous tasks and do not consider the preference-matching problem in the case of heterogeneous tasks.

Therefore, there is an urgent need to study the problem of finding a heterogeneous task assignment that satisfies both the perceived quality preferences of task requesters and the profit preferences of workers under the coordination of different groups in order to accomplish task assignment under group wisdom perception. The intrinsic goal is to find a match such that each of the users is satisfied with their preference assignment, while reducing the communication cost among all recruited users. Therefore, in this paper, we design a stable heterogeneous task assignment algorithm based on stable matching under budget constraints to solve the heterogeneous task assignment problem under group socialization by considering users’ preferences and intergroup cooperation.

## 3. System Model

This section describes a scene in which an MCS platform publishes multiple heterogeneous tasks. The compliance used in this paper is summarized in Table 1. The platform contains N heterogeneous tasks and M users, and assumes the presence of perceptual resources of type L in the system, i.e., $L=\left\{1,\;2,\ldots ,\;L\right\}$. The set of heterogeneous tasks is denoted as $T=\left\{t_{1},\;t_{2},\ldots ,\;t_{N}\right\}$, $S=\left\{s_{1},\;s_{2},\ldots ,\;s_{M}\right\}$ denotes the set of users involved in the task, and it is assumed that user $s_{i}$ has at most $r_{0}$ type of perceptual resources and user $s_{i}$ has perceptual resources denoted as $r(s_{i})=\left\{1,\;2,\ldots ,\;r_{0}\right\},\;r_{0}<L$. Therefore, it is important to recruit a sufficient number of users to participate in the perception task in order to complete the perception task. However, task requesters usually have a limited budget, which limits the number of recruited participants to represent the task’s budget $B_{k}$$t_{k}$, i.e., the bids (benefits) of all users who complete the task $t_{k}$, not exceeding the budget $B_{k}$. In order for users to obtain nonnegative utility, users $s_{i}$ cannot be paid less than their perceived cost. Therefore, each task requires different perceptual resources, e.g., an environmental awareness task requires air pollutant concentration, while a traffic monitoring task requires the speed and location of a car, and the set of perceptual resources required for the task is denoted by $r(t_{k})$$t_{k}$.

As users under group socialization, the study of interuser relationships in quasi-static scenarios can be obtained from the social network of the undirected social graph, where the social relationships between users are represented in the undirected social network $SN=\left(U,E\right)$, where each edge $E_{ij}$, represents a user $s_{i}$, with a user $s_{j}$, with social relationships. Moreover, when users historically perform perceptual tasks, social relations between users can be obtained through cooperative relationships, and the communication cost between users can be calculated as a monotonically increasing function of communication distance [35,36,37]. The communication cost between users affects not only the quality of task cooperation but also the choice of intergroup cooperation; therefore, the intragroup communication cost is defined as the communication cost between the group leader and all group members. Based on the above, different social relationships are organized into different groups $G_{k}$ (group leader is $s_{k}$) and each group can perform a subtask, intragroup communication cost is defined as $T_{k}^{S}$$O_{k}^{G}$ and intergroup communication cost is defined as $I_{k}^{G}$. The sum of intergroup communication costs for collaborating on a task is defined as:

(1) $$W^{G}=\sum_{G_{k}\in G}I_{k}^{G}$$

In general, different perceptual tasks running on different smart terminals will obtain different perceptual quality due to the variability of smart terminals in hardware configuration or perceptual location. The perception cost j is represented by the perceptual resources q(i, j, k) provided by the user $s_{i}$ while performing the perceptual task $t_{k}$, and the perceptual resources provided by the smart terminal for the task denoted by $r(i,\;k)=r(s_{i})\cap r(t_{k})$, then $q(i,\;k)=\sum_{j\in r(i,k)}q(i,\;j,\;k)$ denotes all the perceptual qualities provided by the user $s_{i}$ while performing the perceptual task $t_{k}$. The perceptual quality can be quantified by the coverage of the target area, the time to complete the task, etc. For each smart terminal, users may incur different perceptual costs when engaging in different tasks due to the diversity of tasks and the heterogeneity of resource requirements. Using c(i, j, k) to denote the perceived resource cost j contributed by the user $s_{i}$ in performing the perceived task, then $t_{k}$$c(i,\;k)=\sum_{j\in r(i,k)}c(i,\;j,\;k)$ denotes the total cost $t_{k}$ corresponding to the user $s_{i}$ in performing the task. In addition, p(i, k) represents the reward obtained by the user $s_{i}$ for participating in the task, while $t_{k}$$p_{\max}(i,\;k)$ represents the maximum benefit obtained by the user $s_{i}$ from the task $t_{k}$. The above information is known only to the user and not to other users. In this case, the fairness of the competition is ensured and the privacy of both buyers and sellers is protected to some extent.

**Figure 1:**
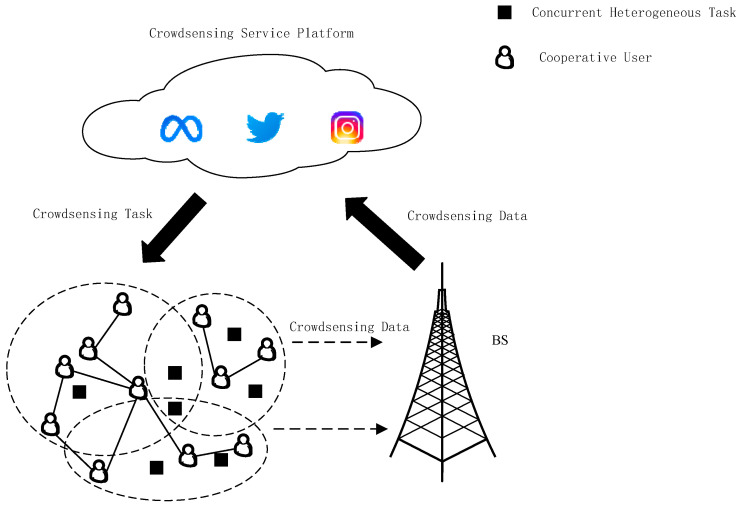
Heterogeneous task assignment model.

The system model takes into account that each user can participate in multiple heterogeneous tasks, as shown in Figure 1.

In addition, an open competition system is considered that allows each user to dynamically adjust the perceived bid after each failed task assignment to compete for more tasks. To capture the interaction between task requestors and users, the model constructs a distributed many-to-many resource transaction model in which task requestors act as buyers of resources and users as sellers of resources. For simplicity, buyers and sellers are used to exchange with tasks or task requesters and users or smart terminals, respectively. After the buyer initiates a demand, the interaction between the buyer and the seller is sequential, as described below.

(1) In each round, each user, as a seller, sends a bid package to interested buyers, which includes the bid and the perceived quality of the executed task.
(2) After the buyers collect the bid packages from the sellers, each buyer selects the local best of the temporarily accepted sellers within its budget constraint. Then, the buyer sends its local decision to the selected seller and rejects the other sellers.
(3) Upon receipt of the buyer’s decision, if the seller is rejected by the buyer, the seller will decide in the next round of trading whether to lower its offer (b). If the seller is accepted by the buyer, its offer remains unchanged in the next round of trading.
(4) Continue the above process until all sellers’ bid packages are accepted by their interested buyers, or all sellers are no longer able to lower their bids.

Based on the above distributed interaction model, the buyer eventually negotiates and completes the transaction with the corresponding seller.

Before the problem is formed, the following variables are first defined.

$w(s_{i})$: The seller’s purchaser $s_{i}$, where $w(s_{i})\in T$.

$w(t_{k})$: Purchasers of $t_{k}$ are recruited by users of $w(t_{k})\in S$.

p(i,k): Buyer $t_{k}$ to Seller $s_{i}$, also expressed as Seller $s_{i}$ from Buyer $t_{k}$.

r(i,k): Perceptual resources provided by Seller $s_{i}$ to Buyer $t_{k}$, where $r(i,\;k)\in r(s_{i})$.

Use $P_{i}=\{p(i,\;k)|\forall t_{k}\in T\}$ for the revenue earned by the seller $s_{i}$ for participating in the task, then $\mathbb{P}=\cup_{s_{i}\in S}P_{i}$ for the revenue earned by all sellers, and ${\mathbb{P}}_{-i}=\mathbb{P}\backslash P_{i}$ for the total revenue earned by all sellers except $s_{i}$ sellers.

Defining the utility of buyers and sellers, for each buyer $t_{k}\in T$, the perceived quality is mainly maximized by the recruited sellers $w(t_{k})$, however, due to budget constraints, the utility of buyers $t_{k}$, can be defined as $U_{T}^{k}$:

(2) $$U_{T}^{k}=\sum_{s_{i}\in w\left(t_{k}\right)}\sum_{j\in r(i,k)}q(i,\;j,\;k)$$

Thus, the goal of $t_{k}$ is:

(3) $$\begin{array}{l} P1:\max_{w(t_{k})}\begin{array}{ll} & U_{T}^{k}(t_{k},\;w(t_{k}),\;\mathbb{P}) \end{array}\\ s.t.\begin{array}{ll} & C1:\begin{array}{ll} & \sum_{s_{i}\in w\left(t_{k}\right)}p(i,\;k)\leq B_{k} \end{array} \end{array} \end{array}$$

where Equation (3) indicates that the total revenue of the sellers recruited by the buyer should not exceed the buyer’s budget, which ensures the buyer’s personal reasonableness. Once a user $s_{i}$ participates in a task $t_{k}$, the perceptual data required for the task is collected and uploaded $t_{k}$. It is assumed that each task has a specific AoI [38] (area of interest, AoI), i.e., the area where the task collects perceptual data. The quality of the perceptual data required for the user $s_{i}$, to provide the task $t_{k}$, is related to the distance from the user to the task and the performance of the device. It is assumed that the quality of the user in the region to complete the task is only related to the performance of the smart device, while the quality of the data outside the AoI region is lower than that of the person in the region and decreases with increasing distance. Specifically, for any $s_{i}\in S$ and $t_{k}\in T$, if user $s_{i}$ is involved in the task $t_{k}$ and user $s_{i}$ is involved in the task $t_{k}$, then the perceived quality of q(i, j, k) provided by j is defined as:

(4) $$q(i,\;j,\;k)=\left\{\begin{array}{c} \lambda_{i},\;D_{i,k}\leq D_{k}\\ \lambda_{i}-\frac{D_{i,k}-D_{k}}{D_{\max}-D_{k}},\;D_{k}<D_{i,k}\leq D_{\max}\\ 0,\;D_{i,k}>D_{\max} \end{array}\right.$$

Since each smart device has different sensing and computing capabilities, not all participants can provide the same level of perceptual quality. $\lambda_{i}$ is the smart device coefficient, which is related to the performance of the device. $D_{i,\;k}$ is the Euclidean distance between the user and the task, $D_{k}$ is the radius of the AoI region, and $D_{\max}$ is the farthest distance to perform the task.

For each seller $s_{i}\in S$, the goal is to maximize the net revenue earned by participating in the task, i.e., the total bids ultimately earned minus the total perceived cost. The user $s_{i}$, whose utility is denoted by $U_{S}^{i}$, is represented as follows.

(5) $$U_{S}^{i}=\sum_{t_{k}\in w\left(s_{i}\right)}\left(p(i,\;k)-c(i,\;k)\right)$$

Thus, the goal of $s_{i}$ is:

(6) $$\begin{array}{l} P2:\underset{w(s_{i})}{\max}\begin{array}{ll} & U_{S}^{i}(s_{i},\;w(s_{i}),\;P_{i}) \end{array}\\ s.t.\begin{array}{ll} & C2:\begin{array}{ll} & c(i,\;k)\leq p(i,\;k)\leq p_{\max}(i,\;k) \end{array} \end{array}\\ \begin{array}{ll} & \;\;C3:\begin{array}{ll} & w\left(s_{i}\right)\subseteq T \end{array} \end{array}\\ \begin{array}{lll} & & \;\;\;\;C4:\begin{array}{ll} & \sum_{j\in r(i,\;k)}j\leq r(s_{i}) \end{array} \end{array} \end{array}$$

The goal is governed by three equations, where the seller’s cost is lower than the seller’s bid and lower than the initial bid, which ensures the individual rationality of the user. The user can only participate in the tasks that exist in the system, indicating that the sum of perceptual resources provided by the user should not exceed the perceptual resources they have.

When buyers collect bidding packs, each buyer chooses the temporarily accepted seller under a budget constraint, which is the local optimum. This problem can be described as the 0–1 backpack problem, which is a typical NP-hard, the capacity of the backpack is the budget of the task $t_{k}$$B_{k}$, and suppose the task $t_{k}$ recruits n users, then the bid of n users is $p_{k}=(p(1,k),\;p(2,k),\ldots ,\;p(n,k))$, the perceived quality of n users is $q_{k}=(q(1,k),\ldots ,\;q(m,k))$, and the user’s choice is determined by the n metavariable $x=(x_{1},\;x_{2},\ldots ,\;x_{n}),\;x_{i}\in \{0,\;1\},\;1\leq i\leq n$, where $x_{i}=1$ denotes the selection of this seller and $x_{i}=0$ denotes the rejection of this seller.

Thus, the 0–1 backpack problem can be expressed as:

(7) $$\begin{array}{l} P3:\underset{x}{\max}\begin{array}{ll} & \sum_{i=1}^{n}q(i,\;k)x_{i} \end{array}\\ s.t.\begin{array}{ll} & C5:\begin{array}{ll} & \sum_{i=1}^{n}p(i,\;k)x_{i}\leq B_{k} \end{array} \end{array}\\ \begin{array}{ll} & \;\;\;C6:\begin{array}{ll} & x_{i}\in \{0,\;1\}, \end{array}1\leq i\leq n \end{array} \end{array}$$

The essence of solving the 0–1 backpack problem is by solving the variable $\left(x_{1},\;x_{2},\ldots ,\;x_{n}\right)$, while the decision of the variable $x_{i}$ is whether to select a user or not. The 0–1 backpack problem can be solved using dynamic programming (DP). On top of that, it is necessary to ensure that the current user base on the social network can satisfy the requirements of each perceptual task at minimal cost.

(8) $$\min\sum_{G}\left(O^{G}+W^{G}\right)$$

**Definition** **1.** *Mobile crowdsensing task assignment is a mapping between* S *and* T *, where* W *represents the result of matching users and tasks: the following conditions are satisfied.*

① *For any purchase of*$t_{k}\in T$*,*$w(t_{k})\in S$*or*$w(t_{k})=\emptyset$.
② *For any sale at*$s_{i}\in S$*,*$w(s_{i})\in T$*or*$w(s_{i})=\emptyset$.
③ *For any buyer and seller of*$<s_{i},\;t_{k}>$*,*$t_{k}\in w(s_{i})$*, when and only when*$s_{i}\in w(t_{k})$.
④ *For any seller of*$s_{i}\in S$*, if*$w(s_{i})\neq \emptyset$*,*$w(s_{i})=t_{k}$*, then*$t_{k}\in w(s_{i})$*, and*p(i, k)≥c(i, k).
⑤ *For any*$t_{k}\in T$*, if*$w(t_{k})\neq \emptyset$*, then*$\sum_{s_{i}\in w\left(t_{k}\right)}p(i,\;k)\leq B_{k}$.

*Condition③ indicates that in a match, the task and the user are mutually matched; condition④ indicates that in a feasible match, the user is paid no less than his perceived cost for completing the task, i.e., the user’s personal rationality is satisfied; and condition⑤ indicates that in a feasible match, the total payoff for the task does not exceed his budget.*

*In a practical MCS system, task requesters and users are usually rational individuals and thus may discontinue tasks if better options are available to improve their utility. In this case, an effective task assignment mechanism should satisfy certain desirable properties, including individual rationality, fairness, non-wastefulness, and stability [39], defined as follows.*

**Definition** **2.** *A match between a buyer* $t_{k}$ *and a seller* $s_{i}$ *is reasonable if*

① *Each seller* $s_{i}$ *, at* $w(s_{i})$ *, to achieve a nonnegative utility, i.e., a nonnegative net profit, when the user’s revenue is* $P_{i}$
(9) $$U_{S}^{i}(s_{i},\;w(s_{i}),\;P_{i})\geq 0$$
② *Each buyer* $t_{k}$ *, at* $w(t_{k})$ *, to* ℙ *, to achieve nonnegative utility, i.e., to match the seller.*
(10) $$U_{T}^{k}(t_{k},\;w(t_{k}),\;\mathbb{P})\geq 0$$

*Personal reasonableness ensures that users are not unwilling to perform the tasks assigned to them; personal reasonableness is a fundamental property of resource transactions, but it is different from the maximum utility that the seller wishes to obtain. For example, obtaining nonnegative utility ensures the seller’s personal reasonableness, while achieving maximum utility as defined in Equation (6) is the seller’s goal.*

*To define fairness, this paper introduces the concept of blocking pairs.*

**Definition** **3.** *Given a match* W *and a payment* ℙ *, a seller* $s_{i}$ *and a set of buyers* M *form a blocking pair* $<s_{i},\;M>$ *if there exists a gain* ${\tilde{P}}_{i}$ *and the following conditions are satisfied.*

① *The seller* $s_{i}$ *, when the user is paid* ${\tilde{P}}_{i}$ *, prefers the buyer* $w(s_{i})$ *than the buyer set* M *, when the payoff is* $P_{i}$ *. That is, the following expression is satisfied.*
(11) $$U_{S}^{i}\left(s_{i},\;M,\;{\tilde{P}}_{i}\right)>U_{S}^{i}\left(s_{i},\;w\left(s_{i}\right),\;P_{i}\right)$$
② *Each buyer in the set* M *wants to include sellers in the set of matching sellers* $s_{i}$ *under the payment* ${\tilde{P}}_{i}\cup {\mathbb{P}}_{-i}$ *instead of matching sellers under the payment* ℙ *, even though it must evict some sellers to make room for sellers* $s_{i}$ *under the budget constraint. That is, for any buyer* $t_{k}\in M$ *, there exists a set of sellers* $w^{\prime }\left(t_{k}\right),\;w^{\prime }\left(t_{k}\right)\in w(t_{k})$ *to be evicted, resulting in*
(12) $$U_{T}^{k}\left(t_{k},\;w\left(t_{k}\right)\backslash w^{\prime }\left(t_{k}\right)\cup \left\{s_{i}\right\},\;{\tilde{P}}_{i}\cup {\mathbb{P}}_{-i}\right)>U_{T}^{k}\left(t_{k},\;w\left(t_{k}\right),\;\mathbb{P}\right)$$

*Blocking a match makes the matching result unstable because a seller*$s_{i}$*, can be more profitable by matching the buyer set*M*, instead of the current match set*$w(s_{i})$*. An arbitrary buyer*$t_{k}\in M$*, can improve its perceived quality by evicting other seller sets, or it can improve its perceived quality by recruiting a seller*$s_{i}$.

**Definition** **4.** *Given a match* W *and a payment* ℙ *, a seller* $s_{i}$ *and a buyer set* M *form a wasted pair* $<s_{i},\;M>$ *if* ${\tilde{P}}_{i}$ *exists and the following conditions are satisfied.*

① *The seller* $s_{i}$ *, when the user is paid* ${\tilde{P}}_{i}$ *, prefers the buyer* $w(s_{i})$ *than the buyer set* M *, when the payoff is s* $P_{i}$ *. That is, the following expression is satisfied.*
(13) $$U_{S}^{i}\left(s_{i},\;M,\;{\tilde{P}}_{i}\right)>U_{S}^{i}\left(s_{i},\;w\left(s_{i}\right),\;P_{i}\right)$$
② *Each buyer in the set* M *wants to include the seller* $s_{i}$ *in the set of matching sellers under the payment* ${\tilde{P}}_{i}\cup {\mathbb{P}}_{-i}$ *instead of matching the current matching seller under the payment* ℙ *, but does not need to evict any seller. That is, for any buyer* $t_{k}\in M$ *that meets the following conditions*
(14) $$U_{T}^{k}\left(t_{k},\;w\left(t_{k}\right)\cup \left\{s_{i}\right\},\;{\tilde{P}}_{i}\cup {\mathbb{P}}_{-i}\right)>U_{T}^{k}\left(t_{k},\;w\left(t_{k}\right),\;\mathbb{P}\right)$$

*The waste pair makes task assignment results unstable because the buyer*$t_{k}$*, can recruit another seller*$s_{i}$*, within its budget constraints, to increase its utility, and this recruitment can also benefit the seller*$s_{i}$.

## 4. Grouping Mechanism

This section describes the grouping mechanism, including group leader initialization and group selection.

Since different social relationships have uncertainty for leaders to choose their neighbors, and different social and communication costs of users in the group will lead to different cost–benefits of the group, in order to improve the effectiveness of leader selection, we select users who share as much information as possible with other users in the group social network in terms of both the sensitivity of users in the social network and the diversity of social network leaders. Moreover, selecting users who are more evenly distributed in the group social network will also result in a more even distribution of affected users, avoiding double counting the same communication and increasing the diversity of affected users.

Using the group leader initialization [40] algorithm, the group leader with the largest increase in influence is iteratively selected and the increase in influence of the candidates added to the set of group leaders is denoted as ΔΦ.

(15) $$\Delta \Phi \left(s_{k}\right)=\Phi \left(F^{*}\cup \left\{F_{k}\right\}\right)-\Phi \left(F^{*}\right)$$

where the influence degree of active users is defined as $\Phi \left(s_{i}\right)$ and the increase in influence degree of candidate users added to the leader set L is denoted as ΔΦ, where $F^{*}$ denotes the set of neighborhood points of all users in the leader set L obtained in the previous iteration. New users are continuously added by the group leader initialization algorithm until the number of group leaders reaches a predefined threshold K or the unselected users can no longer increase their influence. Group leaders are iteratively selected and added to L. Specifically, the maximum increase in current influence is calculated and if the current user is able to increase influence, i.e., $\Delta \Phi \left(s_{k}\right)>0$, it is added to L and removed from U. The neighbor nodes of this group leader are added to $F^{*}$ with a predefined threshold of K minus one; otherwise, the user is removed from S.

The specific implementation steps of the algorithm are shown in the following Algorithm 1 and Algorithm 2.


**Algorithm 1:** Initial leader selector Group leader initialization
**Input:**

T,S,F,K


**Output: a set of L⊂S,|L|≤K**

**Step 1: Initialization.**
1. $L-0,F^{*}-0;$
**Step 2.**
2. While K ≠ 0 and S ≠ 0; do3:  k∗ = $\underset{s_{k}\in S\backslash L}{arg\;max}\Delta \Phi \left(s_{k}\right)$4:  if $\Delta \Phi \left(s_{k}\right)>0$, then5:     $L-L\cup \left\{s_{k^{*}}\right\}$6:      $S-S\backslash \left\{s_{k^{*}}\right\}$7:      $F^{*}-F^{*}\cup \left\{F_{k^{*}}\right\}$8:      K = K − 19:   else10:     $S-S\backslash \left\{S_{k^{*}}\right\}$11:  end if12:end while13:return to the leader set. L



**Algorithm 2:** Collaborative group selection
**Input.**

$T,\left\{r_{j}|t_{j}\in T\right\},F_{k},c^{S},I^{S}$


**Output: a group of users**

$G_{k}$


**Step 1: Initialization.**
1: $G_{k}-\emptyset ,Q^{*}-\emptyset$
**Step 2.**
2: $G_{k}-G_{k}\cup s_{k}$3: for $t_{j}\in T\cap Q_{k}$ do4:      $r_{j}-r_{j}-1$5:      if $r_{j}=0$, then6:          $Q^{*}-Q^{*}\cup t_{j}$7:          $T-T\backslash \left\{t_{j}\right\}$8:      end if9: end for10:while T≠∅; and $F_{k}\neq \emptyset$; do11:    for $s_{i}\in F_{k}$ do12:        if $Q_{i}{}^{s}\cap T=\emptyset$, then13:             $F_{k}-F_{k}\backslash \left\{s_{i}\right\}$14:        else15:            calculate $\Lambda_{i}^{S}$16:        end if17:    end for18:    $s_{i}\in F_{k}$18: For all $\Lambda_{i}^{S}$, sort in a non-decreasing way.19:    $s_{i^{*}}$— has the least number of users $\Lambda_{i}^{S}$20:    $G_{k}-G_{k}\cup s_{i^{*}}$21:    $F_{k}-F_{k}\backslash s_{i^{*}}$22:    for $t_{j}\in T\cap Q_{i^{*}}$ do23:        $r_{j}-r_{j}-1$24:        if $r_{j}=0$, then25:             $Q^{*}-Q^{*}\cup t_{j}$26:            $T-T\backslash \left\{t_{j}\right\}$27:        end if28:    end29: end while30: return the user group: $G_{k}$


The selected group leader needs to find dependent neighbors in the intragroup social network to form a group to complete heterogeneous multitasks in order to achieve good cooperation among users and minimize the communication cost between the group leader and neighbors. First, the group leader $s_{k}$, selected by the group leader initialization algorithm, is added to $G_{k}$ to calculate the current uncompleted tasks and remove the tasks from T that already satisfy the demand. The unsuitable users are filtered out at, and $F_{k}$$\Lambda^{S}$ is computed for the qualified users.

(16) $$\Lambda_{i}^{S}=\frac{\alpha C_{i}^{S}+\left(1-\alpha \right)I_{iK}^{S}}{\left|Q_{i}^{S}-Q^{*}\right|}$$

where the cost-effectiveness of the user is defined as the average cost of covering the outstanding tasks, $\Lambda_{i}^{S}$ is the current cost-effectiveness of the user $s_{i}$, where $C_{i}^{S}$ denotes the social cost of the user and $s_{i}$$I_{iK}^{S}$ denotes the communication cost between the user $s_{i}$ and the group leader. $s_{k}$$Q^{*}$ denotes all perceived tasks completed by the user in the task cooperative group, $\left|Q_{i}^{S}-Q^{*}\right|$ denotes the number of incomplete task sets in the task cooperative group, and the parameter $\alpha \in \left[0,1\right]$ denotes the trade-off between the social cost of the user and the communication cost between the user and the group leader $F_{k}$ The users in $\Lambda^{S}$ are sorted in nondescending order. The smallest user is selected and added to $G_{k}$, and removed from $F_{k}$. When there are no more outstanding tasks or no more neighbors to choose from, the algorithm returns to the user group $G_{k}$.

## 5. Heterogeneous Multitasking Algorithm

In this section, we focus on designing an adjustable bid task allocation (ABTA) algorithm and analyze it theoretically.

ABTA algorithm for heterogeneous multitask assignment.

Each user in an MCS system has different characteristics. In other words, they need different ways of remuneration due to their different perceived resources. Traditional DA algorithms [41] can produce stable matches for the universal admission problem, but the algorithm becomes unstable for the heterogeneous and heterogeneous multitask assignment problem in MCS. In addition, users allow each seller to gradually adjust their bids to the buyer in order to win in the competition. In this case, buyers’ and sellers’ preferences are not fixed in each round. In addition, the number of sellers accepted by each buyer is not predetermined due to the buyer’s budget constraints. To solve these problems, we will introduce a task assignment algorithm based on stable matching. Unlike the traditional DA algorithm, sellers have “one chance to choose” and sellers can submit bids multiple times and resubmit the updated bids to previously rejected buyers.

This section uses $\delta_{i}$ to denote the seller’s $s_{i}$ reduction rate. In other words, each seller $s_{i}$, as long as the utility is nonnegative, can reduce its bid by $\delta_{i}$ after each rejection by the buyer. Assume that $p^{m}(i,\;k)$$s_{i}$, representing the seller, has $p^{1}(i,\;k)=p_{\max}(i,\;k)$, initially set locally by the seller $s_{i}$, relative to the first round $t_{k}$ of the task m. Since users select the tasks they participate in locally, they are not aware of other people’s bids and all interactions take place between local sellers and buyers. In this way, each seller is unaware of everyone else and each buyer is only aware of the bid packages they are interested in. This chapter uses $w^{m}(s_{i})$ to denote the set of buyers to whom $s_{i}$ will send bid packages in the m round, and $w^{m}(t_{k})$ to denote the set of sellers to whom $t_{k}$ will temporarily accept in the m round. The ABTA algorithm operates as follows.

In the first m round, each seller $s_{i}$, selects a buyer in $w^{m}(s_{i})$ and first calculates c(i, k) and then based on $w^{m}(s_{i})=\left\{t_{k}|p^{m}(i,k)\geq c(i,k),\;\forall t_{k}\in T\right\}$r(i, k). Then, the calculation is calculated based on q(i, k)r(i,k) and finally $s_{i}$ sends a bid package to each buyer in $t_{k}$$w^{m}(s_{i})$, where the bid package is the user’s bid $p^{m}(i,\;k)$ and perceived quality q(i, k).

After collecting the sellers’ bid packages, the buyer $t_{k}$, selects the appropriate subset of sellers $w^{m}(t_{k})$, and solves for the best overall perceived quality using the DP algorithm under the budget constraint, i.e., maximizing their utility. Then, the buyer $t_{k}$, temporarily accepts the appropriate sellers $w^{m}(t_{k})$, and rejects the other sellers.

Upon receipt of buyer’s $t_{k}\in w^{m}(s_{i})$ decision, seller $s_{i}$ has the following two options.

If $s_{i}$ is rejected by $t_{k}$ and the current paid $p^{m}(i,\;k)$ is not less than its cost c(i, k), then the bid for $t_{k}$ will be reduced in the next round, i.e., $p^{m+1}(i,\;k)=\max\left\{p^{m}(i,k)-\delta_{i},\;c(i,k)\right\}$.

If $s_{i}$ is accepted by $t_{k}$, or if the current payment $p^{m}(i,\;k)$ is equal to c(i, k), the bid for $t_{i}$ remains the same, i.e., $p^{m+1}(i,\;k)=p^{m}(i,\;k)$.

Use $flag_{i}=0$ to indicate that the seller’s $s_{i}$ bid in this round is the same as the previous round, and vice versa to indicate a different bid. If all users have the same bid as in the m round, the transfer will be terminated at the end of the round, using m $\sum_{s_{i}\in S}flag_{i}=0$ to indicate that the transaction is terminated.

If there are changed bids after the m round, i.e., $p^{m+1}(i,\;k)\neq p^{m}(i,\;k)$, the above process continues in the next round m+1. The detailed steps of the task assignment algorithm with adjustable bids are described below. The specific implementation steps of the algorithm are shown in the following Algorithm 3.


**Algorithm 3:** Stable task assignment algorithm with adjustable bids
**Step 1: Initialization**
1: $m=1,\;\forall s_{i}\in S,\;t_{k}\in T,\;p^{m}(i,k)=p_{\max}(i,k),\;flag_{i}=1$
**Step 2: Buyer and seller’s transaction phase**
2: while $\sum_{s_{i}\in S}flag_{i}>0$ does3: for $s_{i}\in S$ do4:    first calculate $r(i,\;k)=r(s_{i})\cap r(t_{k})$, then calculate c(i, k) and q(i, k) from r(i, k), then calculate $w^{m}\left(s_{i}\right)=\left\{t_{k}|p^{m}(i,k)\geq c(i,k),\;\forall t_{k}\in T\right\}$5:    if $w^{m}\left(s_{i}\right)\neq \emptyset$, then 6:        for $\forall t_{k}\in w^{m}\left(s_{i}\right)$ do7:        the user $s_{i}$, sends the solicitation packages $p^{m}(i,\;k)$ and q(i, k) to the task. $t_{k}$8:        end for 9:    end if10: end for11: for $t_{k}\in T$ do12:    $t_{k}$ after collecting the bid packets sent by sellers, use $w^{m}(t_{k})$ to indicate the set of sellers for the bid packets received at $t_{k}$ use the DP algorithm to select the sellers from $w^{m}(t_{k})$ and reject the unselected sellers13: end for14: for $\forall t_{k}\in T,\;s_{i}\in w^{m}\left(t_{k}\right)$ do15:    if $s_{i}$, is rejected by $t_{k}$ and $p^{m}(i,\;k)>c(i,\;k)$, then16:        $p^{m+1}(i,\;k)=\max\left\{p^{m}(i,\;k)-\delta_{i},\;c(i,\;k)\right\}$17:    else, if $t_{k}$, or $p^{m}(i,\;k)=c(i,\;k)$ receives $s_{i}$, then18:        $p^{m+1}(i,\;k)=p^{m}(i,\;k)$19:    end if20:    $flag_{i}=0$21:    if $p^{m+1}(i,\;k)\neq p^{m}(i,\;k)$, then22:        $flag_{i}=1$23:    end if24: end for25: m=m+126: end while


According to the ABTA algorithm, after a finite number of rounds, the seller’s bid will be accepted or reduced to cost by the buyer, which is the termination condition of the ABTA algorithm. Thus, the algorithm eventually converges in finite time. both buyers and sellers in the ABTA algorithm are individually rational. For each seller $s_{i}$, since it sends bid packages only to buyers whose perceived cost does not exceed the bid price and whose final payment must not be lower than the cost. Therefore, the seller utility achieved by the proposed algorithm is nonnegative. Second, for each buyer $t_{k}$, it will accept a set of sellers within its budget in order to maximize its utility $U_{T}^{k}(t_{k},\;w(t_{k}),\;\mathbb{P})$ in each round. Since the buyer cannot select any user during the matching process, the utility at this point $U_{T}^{k}(t_{k},\;\emptyset ,\;\mathbb{P})=0$, the buyer’s final utility will not be less than 0. In summary, the algorithm achieves individual rationality for both buyers and sellers.

In this paper, we use a counterfactual to prove the fairness of the algorithm. Given the final matching result W, assume that the seller $s_{i}$, when the seller $s_{i}$ forms a blocking pair $\left(s_{i},M\right)$ with the buyer set M, then the seller ${\tilde{P}}_{i}$ has a gain. Then the following two conditions must exist.

(17) $$U_{S}^{i}\left(s_{i},\;M,\;{\tilde{P}}_{i}\right)>U_{S}^{i}\left(s_{i},\;w\left(s_{i}\right),\;P_{i}\right)$$

(18) $$U_{T}^{k}\left(t_{k},\;w\left(t_{k}\right)\backslash w^{\prime }\left(t_{k}\right)\cup \left\{s_{i}\right\},\;{\tilde{P}}_{i}\cup {\mathbb{P}}_{-i}\right)>U_{T}^{k}\left(t_{k},\;w\left(t_{k}\right),\;\mathbb{P}\right)$$

$w^{\prime }\left(t_{k}\right)$ is the subset of sellers that $t_{k}$ excludes from $w(t_{k})$, and since seller $s_{i}$ was not recruited by $t_{k}$ in the ABTA algorithm, its bid at $s_{i}$ must equal its cost in the last round of at $k^{*}$c(i, k). In addition, the buyer $t_{k}$ must prefer the seller in $w(t_{k})$ to the other sellers. Mathematically, the following result can be obtained.

(19) $$p(i,\;k)=p^{k^{*}}(i,\;k)=c(i,\;k)$$

(20) $$U_{T}^{k}\left(t_{k},\;w\left(t_{k}\right),\;\mathbb{P}\right)>U_{T}^{k}\left(t_{k},\;w\left(t_{k}\right)\backslash w^{\prime }\left(t_{k}\right)\cup \left\{s_{i}\right\},\;\mathbb{P}\right)$$

If the buyer $t_{k}$, accepts the seller $s_{i}$ ‘s bid $\tilde{p}(i,\;k)$, then there must be:

(21) $$\tilde{p}(i,\;k)\geq c(i,\;k)=p(i,\;k)$$

Therefore, it is possible to obtain.

(22) $$U_{T}^{k}\left(t_{k},\;w\left(t_{k}\right)\backslash w^{\prime }\left(t_{k}\right)\cup \left\{s_{i}\right\},\;\mathbb{P}\right)\geq U_{T}^{k}\left(t_{k},\;w\left(t_{k}\right)\backslash w^{\prime \prime }\left(t_{k}\right)\cup \left\{s_{i}\right\},\;{\tilde{P}}_{i}\cup {\mathbb{P}}_{-i}\right)$$

where $w^{\prime \prime }\left(t_{k}\right)\subseteq w^{\prime }\left(t_{k}\right)$, can be obtained:

(23) $$U_{T}^{k}\left(t_{k},\;w\left(t_{k}\right),\;\mathbb{P}\right)>U_{T}^{k}\left(t_{k},\;w\left(t_{k}\right)\backslash w^{\prime \prime }\left(t_{k}\right)\cup \left\{s_{i}\right\},\;{\tilde{P}}_{i}\cup {\mathbb{P}}_{-i}\right)$$

Therefore, the matching implemented using this algorithm does not form blocking pairs, and the fairness of the algorithm is proven.

## 6. Simulation Results and Performance Analysis

### 6.1. Experimental Settings

In this section, the performance of the proposed algorithm is evaluated by simulating a scenario of an MCS system using a real dataset [42,43] and by simulating candidate users and their social relationships involved in a perception task using the Gowalla dataset. In this case, a perception task can be defined as sensing traffic information or air pollution levels in a specific area, and mobile users can participate in multiple tasks in a certain area. The values of the simulation parameters are summarized in Table 2. The location of the sensing task is randomly generated from a location close to the mobile smart terminal, and the mobile smart terminal $s_{i}$ can participate in the task $t_{k}$ as long as the distance between $s_{i}$ and $t_{k}$ does not exceed a threshold value $D_{\max}$. The distance of the task from the current location of the smart terminal user is recorded and the execution cost c(i, j, k) is set based on the perceived quality criteria provided by the user for each task $\left[5,\;10\right]$. The initial maximum bid set for each seller is $\left[15,\;20\right]$, which is higher than the task execution cost, to ensure the individual reasonableness of the seller. To ensure that enough multiple users are recruited for each task, the budget for each task is set at $\left[175,\;275\right]$. The specific parameters are listed below, and all methods were performed on MATLAB 2019a.

### 6.2. Performance Indicators

For each simulation, examples from the dataset were selected for simulation and the following indicators were compared and analyzed.

1. Reward/Cost. This indicator ensures nonnegative personal utility for the user by comparing the reward under matched outcomes to the cost of performing the task.
2. Budget/Total Payment. This indicator ensures that task personalization is not a negative utility by comparing the task budget and its total payment to the user under the task matching outcome.
3. System Utility (Task Service Quality) with different number of users: this indicator evaluates the performance of the number of users in each algorithm in optimizing the system utility, which is verified by the gap between the algorithm and the system optimal solution.
4. System Utility (Task Service Quality) with different number of tasks: this indicator evaluates the performance of the number of tasks in each algorithm in terms of optimizing the system utility, which is verified by the gap between the algorithm and the system optimal solution.

### 6.3. Baseline Approaches

For comparison purposes, three comparison algorithms are introduced, namely the algorithm for optimal system efficiency, the ASTA algorithm, and the greedy task assignment (GTA) algorithm.

System Utility-Optimal algorithm: find a match that maximizes the quality of service for the buyer. In this simulation, the system utility-optimal task assignment problem is transformed into a 0–1 integer linear programming problem and solved using MATLAB.

ASTA [44] (approximate alliance stable task allocation, ASTA) algorithm: approximate the alliance stable task allocation algorithm that considers a system model similar to this paper and where the buyer has heterogeneous resource requirements.

GTA [45] greedy algorithm. A bipartite graph is built based on a list of user and task preferences. The GTA algorithm always chooses the match with the highest weight value under budget constraints in the best way and updates the budget of the task for each selection until all edges are matched.

In the ASTA algorithm, the worker makes a matching request to the task in the order of the preference list, and the requested task either accepts the worker directly as the budget allows, or optimizes the current matching result by solving the 0–1 backpack problem. The GTA algorithm, on the other hand, has the longest running time, which is consistent with its time complexity being the highest among the three algorithms. Within all the algorithms, our proposed algorithm has the highest percentage of stable matching pairs, and the GTA algorithm does not take into account the stability of matching.

### 6.4. Complexity Analysis

The grouping mechanism of the GOABTA algorithm is based on leader initialization, and since the computation of the while-loop is at most $O\left(S\right)$, its time complexity is $O\left({\left|S\right|}^{2}\right)$.In Collaborative group selection, the computational overhead of computing the current outstanding task is at most $O\left(\max\left\{\left|Q_{i}^{S}\right|:s_{i}\in G_{k}\right\}\right)$, and the total complexity of the for loop is at most $O\left(\left|S\right|\cdot \log\left|S\right|\right)$ for each $F_{k}\subseteq S$, since there are $F_{k}$ users connected by leader $s_{k}$. The task assignment algorithm converges in finite time, and the termination condition of the algorithm is reached only after finite rounds of transactions in which the seller’s bid will be accepted by the buyer or reduced to cost. Thus, the algorithm eventually converges in finite time and achieves individual rationality for both buyers and sellers.

### 6.5. Result Analysis

#### 6.5.1. Personal Rational Analysis

**Figure 2:**
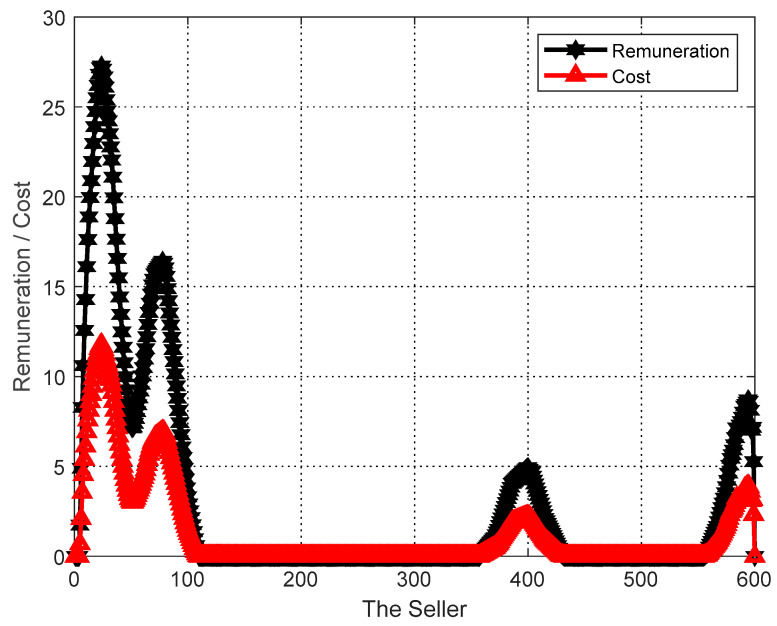
Personal analysis of the user.

**Figure 3:**
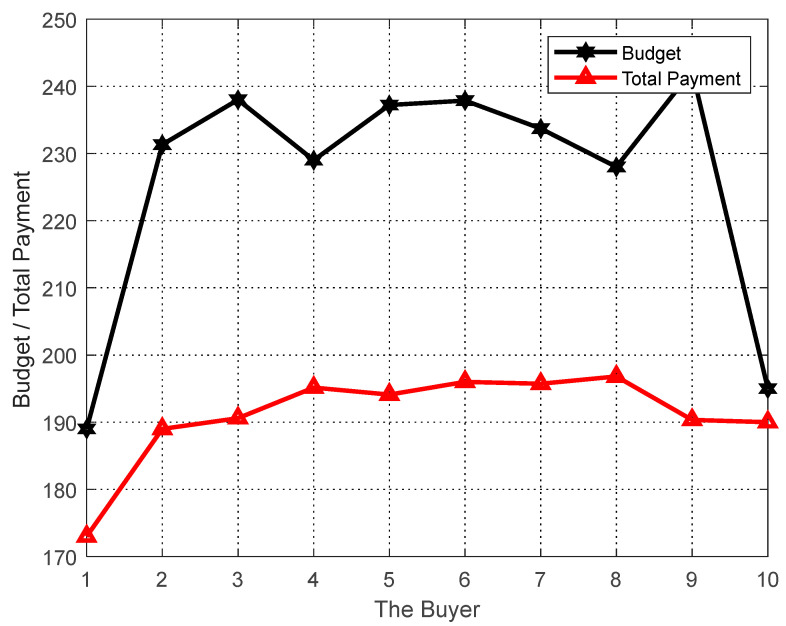
Individual analysis of the task.

The performance of the proposed algorithm is evaluated in terms of achieving personalization of users and tasks. In the simulation, the sellers $s_{i}$, i.e., $\delta_{i}=1$, the number of sellers M=600 (for selecting users in group social relations), the number of buyers N=10, and the reduction rate of sellers is set to 1. The final matching result gives the final payoff of the users and their cost to perform the task. As shown in Figure 2, each user ends up with a payoff no less than the cost of completing the task, ensuring a nonnegative utility for each seller. In addition, the final task matching results give the budget for the task and the total cost paid to the users, and as can be seen in Figure 3, the total cost paid to the buyers at the end of the algorithm does not exceed their budget, thus achieving a nonnegative utility for each buyer. That is, the proposed task allocation algorithm ensures the individual rationality of both buyers and sellers.

As can be seen in Figure 2 and Figure 3, the GO-ABTA algorithm always ensures the individual rationality of users and tasks during the matching process. During the matching process, the total payment for the task is always lower than the task budget (the minimum payment for completing the task), and the trend of the total payment curve is the same as the trend of the task budget curve. In grouped social relations, the user’s payment is always higher than the cost of performing the task (only the selected user performs the task in the grouping mechanism), and the trend of the payment curve is the same as the trend of the task execution cost curve.

#### 6.5.2. System Utility Analysis

**Figure 4:**
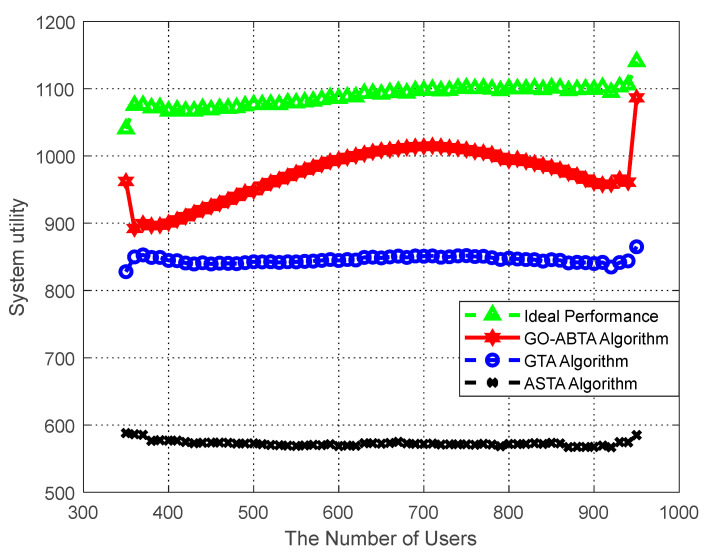
Performance of system utility compared to number of users.

**Figure 5:**
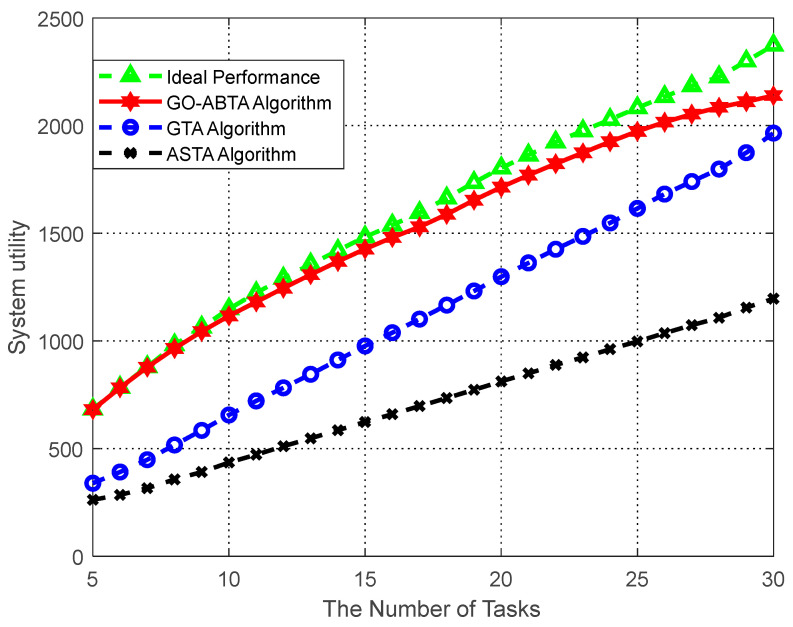
Performance comparison of system utility versus number of tasks.

The performance of the proposed algorithm in optimizing the system utility is analyzed. In the simulation, the number of buyers is fixed at 15, while the number of sellers varies from 350 to 950, as shown in Figure 4, and it can be seen that the algorithm in this paper has the smallest gap with the optimal solution of the system. The stability of the system cannot be guaranteed because the optimal solution of the system efficiency only aims at maximizing the utility of buyers and ignores the individual tasks and users’ preferences. In the simulation, the number of sellers is fixed at 1200, while the number of buyers varies from 5 to 30, as shown in Figure 5, which shows that the gap between the algorithm in this paper and the system optimal solution is minimized. The simulation results also show that the algorithm outperforms the ASTA algorithm and the GTA algorithm. In addition, it can be seen from the figure that when the number of sellers increases, the utility of buyers increases in all four cases. This is because the more sellers there are, the more buyers can recruit enough sellers to obtain higher utility.

Figure 4 and Figure 5 show the advantages of the GO-ABTA algorithm for different numbers of users and tasks. In Figure 4, it is clear that the GO-ABTA algorithm outperforms the GTA algorithm and the ASTA algorithm (the gap between the optimal solution and the system is the smallest), and the task utility increases for all four curves as the number of users increases. From Figure 5, it can be seen that the GO-ABTA algorithm is closest to the gap between the optimal solution and the system, and the task utility under all four curves improves significantly as the number of tasks increases. the main reasons for the best performance of the GO-ABTA algorithm are: (1) the GO-ABTA algorithm achieves buyer and seller after a limited round of transactions, and the seller’s offer is accepted or reduced to cost by the buyer, achieving individual rationality; (2) as the number of tasks and users increases, the grouping of users under group social relations brings more budget and more users to choose from, thus increasing the number of interaction rounds and enabling tasks to recruit more compatible users and achieve higher utility.

Therefore, it can be concluded that the GO-ABTA algorithm is superior to the GTA and ASTA algorithms and is closest to the system optimal solution.

## 7. Conclusions

With the rapid development of mobile internet and smart devices, mobile crowdsensing has become a widely used data collection method in smart cities, and reasonable task assignment is always essential in mobile crowdsensing. In this paper, an adjustable bidding task assignment method is used to describe the assignment of perceptual tasks in MCS systems. To address the problem of optimizing only the performance of the global system in real mobile groupwise perception scenarios while ignoring user preferences and affecting future user participation, the heterogeneous task assignment problem is further investigated in conjunction with user preferences. In addition, the interaction between task requesters and users is described as a many-to-many matching problem under group social relations by a distributed many-to-many resource transaction model. First, the group selection of users is performed through the grouping mechanism of group leader initialization and group selection, and then the task assignment process is described based on stable matching to complete a stable heterogeneous task assignment algorithm under budget constraints. The simulation results verify the individual rationality, stability and convergence of the GO-ABTA algorithm and compare the effectiveness and superiority with other algorithms. In the future, we will still focus on the direction of task assignment algorithms in complex heterogeneous resource scenarios. We will extend GOABTA through game theory and preference matching to improve task-aware quality, ensure platform revenue and increase user revenue. The research in this paper can be reviewed as a reference for MCS-related research.

## Figures and Tables

**Table 1:** Symbols of model parameters.

Symbols	Description
T	Task Settings
S	User Group Settings
L	Perceptual Resource Set
$r_{s_{i}}$	User’s perceived resources $s_{i}$
$B_{k}$	Mission budget $t_{k}$
$r_{t_{k}}$	Task $t_{k}$ Required Perceptual Resources
$G_{k}$	Group k in the population
$s_{k}$	Group leader in the group $G_{k}$
$T_{k}^{S}$	A subset of the executable tasks in the group k
$O_{k}^{G}$	Intra-group exchange costs for the k group in the population
$I_{k}^{G}$	Intergroup exchange costs for the k group in the population
$W^{G}$	Communication costs between collaborative groups
q	The perceived quality provided by the user performing the task
c	Perceived costs incurred by users when performing tasks
p	Pay for tasks performed by users
λ	Equipment factor
$p_{\max}$	Maximum reward for users from the task
$U_{T}^{k}$	Utility of the assignment $t_{k}$
$\delta_{i}$	Bid reduction rate
$I_{iK}^{S}$	Communication costs between the user $s_{i}$ and the group leader $s_{k}$
$Q^{*}$	Completion of tasks by users in task collaboration groups
$p^{m}$	Sellers bid at m, relative to the task.
$w^{m}$	A group of buyers will send a bid package in round m.
SN	Non-directional social networks
j	Perceived quality provided by the user during the execution of the task $s_{i}$ $t_{k}$
$F^{*}$	The set of neighbor nodes of the current group leader
$\Lambda^{S}$	The current cost effectiveness of the user
$C_{}^{S}$	The social cost of the user

**Table 2:** Main simulation parameters.

Parameters	Value	Description
L	5–30	Types of perceived resources in MCS
$r_{0}$	3–5	Types of perceptual resources owned by the terminal
c(i, j, k)	5–10	Cost of user participation in tasks $s_{i}$ $t_{k}$
$B_{k}$	175–275	Mission budget $t_{k}$
$p_{\max}(i,\;k)$	15–20	The user $s_{i}$, participated in the highest bid for the task $t_{k}$
$\lambda_{i}$	6–10	Equipment factor

## Data Availability

Data sharing not applicable.
